# Whole mitochondrial genome of a Geoffroy’s Rousette, *Rousettus amplexicaudatus* (Pteropodidae)

**DOI:** 10.1080/23802359.2019.1676671

**Published:** 2019-10-12

**Authors:** Rogel Victor D. Mendoza, Ian Kendrich C. Fontanilla

**Affiliations:** a DNA Barcoding Laboratory, Institute of Biology, College of Science, University of the Philippines, Quezon City, Philippines;; b Philippine Genome Center, University of the Philippines, Quezon City, Philippines

**Keywords:** Chiroptera, Pteropodidae, Rousettus, whole mitogenome

## Abstract

The whole mitochondrial genome assembly of *Rousettus amplexicaudatus* belonging to the Pteropodidae found in the Philippines was sequenced and characterised. *De novo* sequence assembly yielded a 16,509bp sequence with an overall base composition of 32.43% A, 25.39% T, 26.17% C, and 14.02% G. The mitochondrial genome is composed 22 tRNA genes, 13 protein-coding genes, and two rRNA genes. Molecular phylogeny of the order Chiroptera based on the maximum likelihood and Bayesian inference trees supports the classification of *R. amplexicaudatus* to genus *Rousettus* and family Pteropodidae. Furthermore, both trees support the modern division of order Chiroptera into the Yinpterochiroptera and Yangochiroptera clades.

The species *Rousettus amplexicaudatus* (Geoffroy’s Rousette) is a member of the family of fruit bats (Pteropodidae) widely distributed in the Southeast Asian region and is considered of ‘Least Concern’ by the IUCN (Csorba et al. 2008). The species also belongs to the only genus of Pteropodidae with an echolocating ability (Hassanin 2014; Ingle and Heaney 1992). However, lack of whole mitogenome sequences of the species poses a problem in understanding evolutionary relationship of bats in the Philippines. In this study, we sequenced and provided the whole mitochondrial genome of *R. amplexicaudatus,* which is the first mitogenome of pteropodid species in the Philippines.

Bat muscle tissue was obtained from the Palanan Permanent Forest Dynamic Plot in Palanan, Isabela (17° 2′ 24.756″ N, 122° 23′ 8.088″ E). The specimen tissue with specimen number MRMD 2101 was deposited in the DNA Barcoding Laboratory in the Institute of Biology, University of the Philippines Diliman. DNA extraction was done using a genomic DNA extraction kit (Thermo Scientific, USA), and extracted DNA was sent to the Philippine Genome Centre (PGC) for sequencing using the Illumina MiSeq 500 platform. Forward and reverse reads from high throughput sequencing was used for *de novo* assembly of the whole mitochondrial genome using NOVOPlasty 2.7.2 (Dierckxsens et al. 2017) and was annotated using MITOS (Bernt et al. 2013). Maximum likelihood tree (Felsenstein 1981) with 1000 bootstrap replicates and Bayesian inference tree (Huelsenbeck et al. 2001) with 11 million generations at a heating temp of 0.04 for the posterior probabilities were constructed using IQtree (Nguyen et al. 2015) and MrBayes 3.2 (Ronquist et al. 2012), respectively. Optimal models of substitution for each non-coding gene region and each codon position of protein-coding genes were determined using PartitionFinder (Lanfear et al. 2012).

A 16,509bp sequence of *R. amplexicaudatus* was assembled and submitted to GenBank with the Accession number MN125184. The genome contains 22 tRNA genes, 13 protein-coding genes and two rRNA genes with an overall base composition of 32.43% A, 25.39% T, 26.17% C, and 14.02% G. The Origin of L-strand replication is assumed to be found between genes encoding tRNA^Asn^ and tRNA^Cys^.

All protein-coding genes are transcribed at the light strand except for NADH dehydrogenase subunit 6 (ND6), which is transcribed at the heavy strand. All protein-coding genes have a complete stop codon, except for cytochrome oxidase subunit 3 (cox3) and NADH dehydrogenase subunit 4 (ND4), which are completed by addition of -AA and -A, respectively. All protein-coding genes are terminated by the stop codon TAA, except for NADH dehydrogenase subunit 2 (ND2) and NADH dehydrogenase subunit 6 (ND6), which are terminated by TAG, as well as cytochrome oxidase subunit b (Cytb), which is terminated by AGG. All tRNAs follow the standard cloverleaf structure except for tRNA^Ser(AGY)^, which lacks the Dihydrouridine (DHU) arm.

Phylogenetic analysis using the 22 tRNA genes, 13 protein-coding genes, and two rRNA genes of the Chiroptera whole mitogenome sequences from GenBank show high support in the classification of *R. amplexicaudatus* into the genus *Rousettus* (Bootstrap = 100; PP = 1.00) and family Pteropodidae (Bootstrap = 100; PP = 1.00) (Figure 1). Furthermore, both trees support the modern division of the Chiroptera into Yangochiroptera (Bootstrap = 53; PP = 1.00). and Yinpterochiroptera (Bootstrap = 99; PP = 1.00).

**Figure 1.**
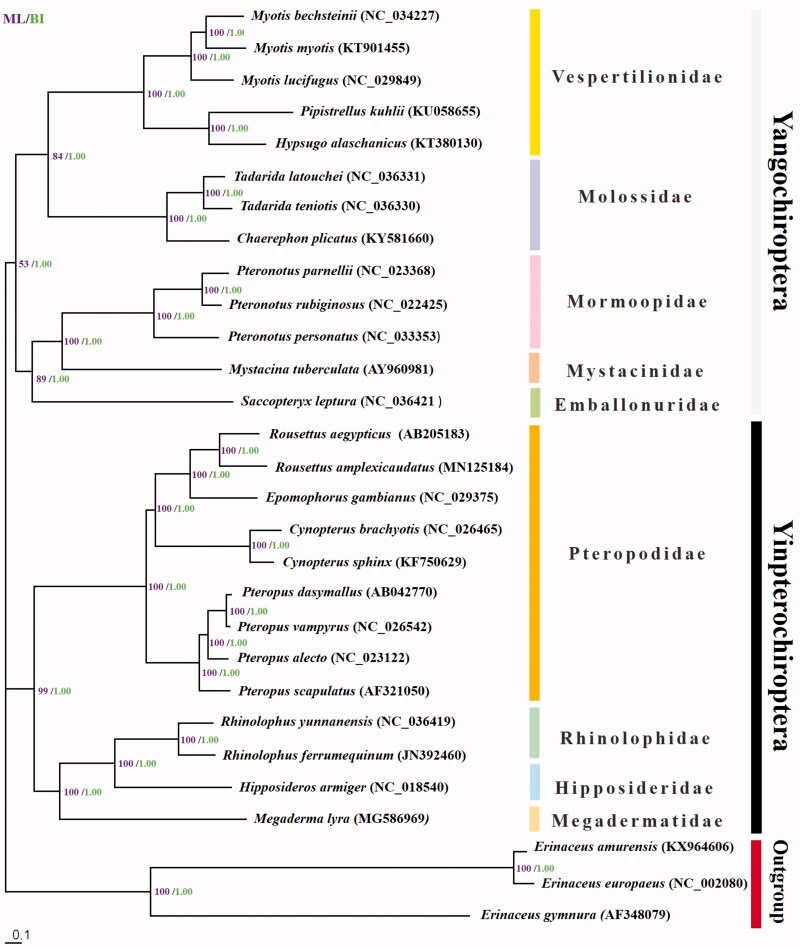
Maximum likelihood (ML) tree of the order Chiroptera based on 14450 nucleotides of the whole mitochondrial genome, with family Erinaceidae (*Erinaceus amurensis, Erinaceus europaeus* and *Erinaceus gymnura*) as the outgroup taxa. Values on the nodes represent percent bootstrap values based on 1000 bootstrap replicates for ML and posterior probabilities based on Bayesian inference (BI); values less than 50% for ML bootstraps and 0.7 for PP are not shown. Scale bar represents one nucleotide substitution for every 10 nucleotides.
